# T-World Virtual Human Cardiomyocyte. II. Organ-Scale Simulations and Applications

**DOI:** 10.1161/CIRCRESAHA.125.328123

**Published:** 2026-04-08

**Authors:** Jakub Tomek, Maxx Holmes, Hector Martinez-Navarro, Xin Zhou, Abdallah I. Hasaballa, Zhinuo J. Wang, Lucas Arantes Berg, Ambre Bertrand, Michael A. Colman, Alfonso Bueno-Orovio, Donald M. Bers, Blanca Rodriguez, Jordi Heijman

**Affiliations:** Department of Anatomy, Physiology and Genetics (J.T.), University of Oxford, United Kingdom.; Department of Computer Science (M.H., H.M.-N., X.Z., A.I.H., Z.J.W., L.A.B., A.B., A.B.-O., B.R.), University of Oxford, United Kingdom.; Department of Pharmacology, University of California, Davis (J.T., D.M.B.).; School of Biomedical Sciences, University of Leeds, United Kingdom (M.A.C.).; Gottfried Schatz Research Center, Division of Medical Physics and Biophysics, Medical University of Graz, Austria (J.H.).; Department of Cardiology, Cardiovascular Research Institute Maastricht (CARIM), Faculty of Health Medicine and Life Sciences, Maastricht University, The Netherlands (J.H.).

**Keywords:** adrenergic agents, arrhythmias, cardiac, calibration, myocytes, cardiac, stroke volume

## Abstract

**BACKGROUND:**

Mechanistic cardiac simulations are increasingly used in research, pharmaceutical development, and regulatory science, yet most existing human cardiomyocyte models lack the generality required for predictive translation across scales. Our recently developed T-World model overcomes this barrier by reproducing all major cellular arrhythmia mechanisms and showing comprehensive agreement with experimental and clinical data. Here, we aimed to demonstrate the utility of T-World for organ-level and translational research, from ionic mechanisms of arrhythmogenesis to emergent whole-heart physiology.

**METHODS:**

T-World was embedded into anatomically realistic models of biventricular electrophysiology and electromechanics derived from clinical imaging for organ-scale simulations. Drug safety was assessed using populations-of-single-cell models exposed to 60 compounds with updated CredibleMeds annotations. Mechanistic drug-efficacy studies on mexiletine were conducted using long QT syndrome type 2 model variants. Disease applications included arrhythmia mechanisms in human type 2 diabetes and the proarrhythmic potential of Na_V_1.8, a neuronal sodium channel ectopically expressed in cardiac disease.

**RESULTS:**

T-World reproduced human-like ECG morphology and ventricular mechanics (ejection fraction of 61%) and generated ventricular fibrillation under physiologically relevant ischemic conditions without parameter tuning. In the drug safety assessment of torsadogenic risk, T-World achieved 87% accuracy and 100% specificity, and exposed incomplete pharmacological descriptions based on in vitro measurements for lidocaine and cilostazol. Mexiletine simulations revealed that both I_NaL_ and I_CaL_ inhibition underlie its antiarrhythmic benefit in long QT syndrome type 2. Cellular simulations of type 2 diabetes remodeling explained heightened vulnerability to early afterdepolarizations and increased risk of alternans associated with diastolic dysfunction, mechanistically linked to SERCA (sarco/endoplasmic reticulum Ca^2+^ ATPase) reduction. Finally, even minor expression of Na_V_1.8 can directly trigger early afterdepolarizations through uniquely right-shifted activation and inactivation properties.

**CONCLUSIONS:**

T-World provides a unified, human-specific open-source platform bridging cellular mechanisms with organ-level dynamics and translational outcomes. Its predictive performance across arrhythmia, contraction, drug safety/mechanisms, and disease physiology makes it a powerful tool for multiscale cardiac research, therapeutic discovery, and next-generation cardiac digital twins.

**Novelty and Significance**

**What Is Known?**

- Mechanistic models of ventricular myocytes are increasingly used for studying drug safety and disease mechanisms.
- The domain of application of existing tools is limited by their lack of generality, as well as the incompleteness of pharmacological or pathophysiological descriptions.

**What New Information Does This Article Contribute?**

- Organ-scale simulations of the T-World human ventricular cardiomyocyte model produce realistic electrocardiograms, ventricular fibrillation under physiologically relevant ischemia, and normal ejection fraction without parameter tuning.
- T-World achieves high accuracy and specificity in predicting drug-induced proarrhythmia and can be used to identify incomplete descriptions of candidate drug effects to guide the collection of additional experimental data.
- The model identifies mechanistic drivers of arrhythmia in type 2 diabetes and demonstrates how even small amounts of neuronal-type sodium channels in disease can surprisingly drive arrhythmogenic behaviors.

Mechanistic cardiac models hold great promise for bridging molecular mechanisms to clinical outcomes, yet most have struggled to translate robustly from single cells to whole-heart physiology. Building on the highly general T-World cardiomyocyte model, this study demonstrates its predictive power across scales and applications. When integrated into anatomically realistic biventricular models, T-World reproduces human-like electrocardiograms, generates ventricular fibrillation under physiologically relevant ischemic conditions without parameter tuning, and supports realistic ventricular mechanics. In drug safety testing, the model achieves high accuracy and perfect specificity for drug-induced proarrhythmia and uniquely identifies incomplete pharmacological descriptions by revealing discrepancies between simulated and known cell-level drug effects. Mechanistic simulations further uncover contributors to arrhythmic vulnerability in type 2 diabetes and show that minimal expression of neuronal sodium channel Na_V_1.8 can directly initiate early afterdepolarizations. Together, these findings establish T-World as a unified, human-specific platform that links ionic mechanisms to organ-level behavior and translational outcomes. This framework advances the development of predictive digital twins and supports more reliable drug evaluation and disease modeling while reducing reliance on animal experimentation.

**Meet the First Author, see p e000754 | Editorial, see Article by Kucera**

Computational modeling and simulations of cardiac cellular and organ physiology increasingly influence real-world applications in the pharmaceutical industry^1^ and regulatory bodies, such as the FDA (US Food and Drug Administration) ^2^ and the European Medicines Agency.^3^ Combined with recent advances in data availability, hardware, and software, these models are driving the vision of the digital twin technology,^4^ producing a virtual tool that integrates clinical data acquired for an individual and enables personalized diagnosis and treatment strategies.

Although multiple successful models of human ventricular cardiomyocytes have been developed to investigate specific mechanisms of cardiac (patho)physiology and arrhythmia, previous model families lacked generality, capturing only a small subset of arrhythmic behaviors. This limits their utility, for example, for analyzing multifactorial drug effects, modeling complex diseases, or integrative arrhythmia studies. In addition, most previous models were unable to robustly bridge the spatial scales from cell to organ, for example, in terms of organ-level arrhythmia inducibility or linking cellular excitation to pump function. This lack of generality partly explains why different cellular models have typically been used to study aspects of arrhythmogenesis at the cellular versus organ level.^5^

The highly general model of the human ventricular myocyte T-World that was introduced in our accompanying article in this journal provides a comprehensive mechanistic representation of electrophysiology, excitation-contraction coupling, β-adrenergic signaling, sex differences, and contraction.^6^ Through a set of key innovations integrated with carefully curated and assembled components from prior state-of-the-art models, it achieves an extensive agreement with human experimental and clinical data, showing strong predictivity in independent validation using data not used in model creation. Crucially, it reproduces, in a single model, all key cellular arrhythmia mechanisms: early and delayed afterdepolarizations, alternans, and steep S1 to S2 restitution.

Building upon this foundation, this article extends the T-World framework from cellular mechanisms to organ-scale and translational levels, demonstrating its capacity to integrate across scales and applications. Whereas the accompanying paper focused on the model’s development, calibration, and validation, here, we explore its predictive and mechanistic utility in realistic whole-organ simulations, pharmacological testing, and disease modeling. We show that T-World reproduces human-like ECG morphology and, crucially, can generate ventricular arrhythmia under physiologically relevant ischemic conditions without parameter tuning. Furthermore, we show that when embedded in an organ-scale model, T-World generates a human-like ejection fraction and pressure-volume (PV) loop. This establishes the model’s suitability for organ-level studies of arrhythmogenesis and contraction, bridging the gap between ionic mechanisms and emergent electrical dynamics in the intact heart.

We then leverage T-World’s predictive capabilities for in silico drug safety and efficacy assessment, an important application with direct industry relevance,^1,7–10^ showing accurate classification of torsadogenic risk. Incomplete drug description data can lead to erroneous prediction of proarrhythmic risk,^11^ and we demonstrate how T-World’s electrophysiological and contractile responses can be leveraged to identify inconsistencies in pharmacological data sets. Finally, we illustrate the model’s value for disease-specific and mechanistic studies, using it to uncover drivers of arrhythmogenesis in type 2 diabetes (T2D)–driven diabetic myocardial disorder and to reveal a potential proarrhythmic role of the neuronal sodium channel Na_V_1.8 in the diseased myocardium. Together, these results position T-World as a unified, data-driven, and mechanistically faithful platform for studying human cardiac electrophysiology, from subcellular dynamics to whole-organ behavior and therapeutic translation.

## Methods

Please refer to the Methods section of the accompanying article for details of model development, calibration, and validation of the T-World cardiomyocyte model.^6^ Detailed methods for organ-scale simulations, drug safety and efficacy analyses, model variants incorporating T2D-related remodeling, and formulation of Na_V_1.8 current are given in the Supplemental Methods and are briefly summarized below.

### Data and Source Code Availability

The T-World cardiomyocyte model is distributed as open-source code and is available at https://github.com/jtmff/TWorld/, including sample scripts demonstrating its functionality. An online graphical user interface enabling running T-World simulations is available at https://t-world-simulator-multipage-production.up.railway.app/. Background of the T-World development, including the description of various dead ends that we encountered during development, will be provided at the blog underlid.blogspot.com.

### Organ-Level Simulations

The T-World cardiomyocyte model was integrated into multiscale computational modeling and simulation frameworks to demonstrate its suitability for organ-level simulations. First, multiscale electrophysiological modeling and simulation were considered with methods described in the study by Martinez-Navarro et al,^12^ including personalization to anatomy and ECG, and the Purkinje system as in the studies by Riebel et al^13^, Camps et al^14^, and Berg et al.^15^ This enabled simulations from ionic dynamics to the ECG in normal and acute regional ischemia. Ischemia-induced ionic alterations were introduced in the T-World model as previously done with ToR-ORd^12^ and applied to an ischemic region, resulting from left-anterior descending artery occlusion. Sinus rhythm was simulated through stimulation of the conduction system as in previous work.^13–15^ Arrhythmia was induced by a stimulation protocol with progressively shorter cycle lengths (more details in the Supplemental Material). Furthermore, the T-World model was introduced in a multiscale electromechanical modeling and simulation framework described in the study by Wang et al^16^ to evaluate its suitability for realistic organ-level simulations of electrophysiology and mechanics, including ejection fraction. Further details are provided in the Supplemental Material.

### Drug Safety and Efficacy Assessment

We generated a population of 1000 cell models for the drug safety assessment study, representing variation in ionic currents and calcium handling, similar to Passini et al.^10^ A subpopulation was selected meeting calibration criteria based on experimentally observed ranges in humans (Table S1). The population was used to test 60 reference compounds with known arrhythmic risk^17^ at multiple concentrations. In addition, the antiarrhythmic effects of mexiletine were evaluated based on 2 experimental data sets (Table S2).

### Simulating T2D

We based our baseline model of T2D predominantly on the most comprehensive human-based data set by Ashrafi et al^18^ and explored additional effects reported in literature by creating 6 model variants reflecting different combinations of late sodium current (I_NaL_), Ca^2+^/calmodulin-dependent protein kinase II (CaMKII), and L-type calcium current (I_Ca,L_) remodeling (Table S3).

### Modeling Na_V_1.8

A separate version of T-World, including the Na_V_1.8 current, was created by adding the Choi-Waxman formulation^19^ to the baseline model.

## Results

### Organ-Scale Simulations of Arrhythmia and Contraction

**Figure 1.**
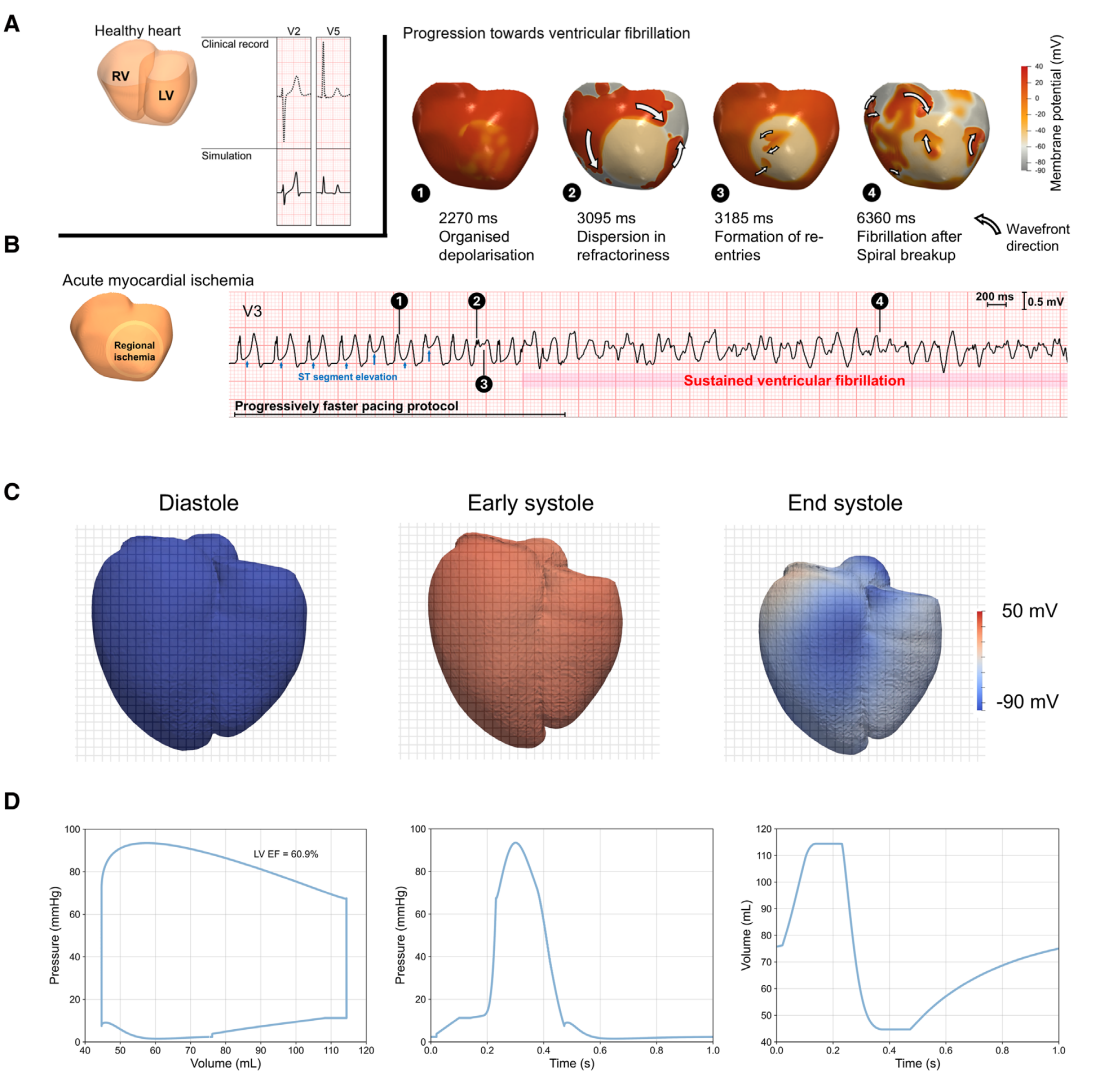
**Organ-level arrhythmia development and contraction. A**, Healthy biventricular model constructed from clinical magnetic resonance imaging data and ECG simulation (solid line) compared with the ECG record from the patient used for the ventricular anatomy. **B**, Ventricular fibrillation simulation in the setting of acute ischemia when the stimulation rate is progressively increased. Heart snapshots above the ECG illustrate the spatial distribution of membrane potentials at different stages of progression toward fibrillation. Examples of action potentials in normal, border, and infarct zones before transition to arrhythmia are given in Figure S1. **C**, Snapshots of a contracting heart built using T-World during (1) diastole, (2) early contraction during electrical activation, and (3) end-systolic contraction. **D**, Corresponding pressure-volume loop, and pressure and volume transients. Videos of transition to ventricular fibrillation and contraction are provided as the Supplemental Material (Videos S1 and S2).

While T-World is a biophysically detailed model capturing a broad range of cardiac cell physiology, it remains highly computationally tractable (ca. 5–10 beats per second on a standard personal computer). This makes it suitable for organ-level studies and reconstruction of the ECG. We assessed the model’s performance across scales by building a 3-dimensional biventricular model based on a patient-specific anatomy, with the simulation yielding an ECG that closely resembled the patient’s ECG signal (Figure 1A).^12,20^ A major application of whole-organ models is the study of arrhythmia, such as ventricular fibrillation, where the impact of tissue-level phenomena such as fibrosis and conduction heterogeneities can be considered. However, numerous advanced models such as ToR-ORd or ORd struggled to produce ventricular fibrillation dynamics, unless their parameters were specifically tuned for this purpose (in addition to imposing a proarrhythmic substrate such as localized ischemia).^12,21^ By contrast, T-World does reproduce ventricular fibrillation in the setting of acute anteroseptal ischemia and progressively increasing rate of stimulation (Figure 1B). Initially, the electrical propagation is stable, only manifesting ST-segment–elevation (a hallmark of acute ischemia), but, as the stimulation rate is increased, reentrant wave fronts appear (Figure 1B, snapshots 2 and 3), and gradually progress to spiral wave breakup and ventricular fibrillation (snapshot 4).

Representation of mechanical force development in T-World, together with recent technological and software advances,^14,22^ enables multiscale simulations of organ-scale contraction. To demonstrate the readiness of T-World for application in contraction studies, we incorporated the T-World model in a multiscale biventricular electromechanical model based on patient magnetic resonance imaging data previously described in the study by Wang et al^^16^^ and the Supplemental Methods. Electromechanical simulations produce appropriate mechanical contraction (Figure 1C and 1D) with an ejection fraction of ≈61%, in line with normal values in humans.

### Drug Safety and Efficacy Assessment

**Figure 2.**
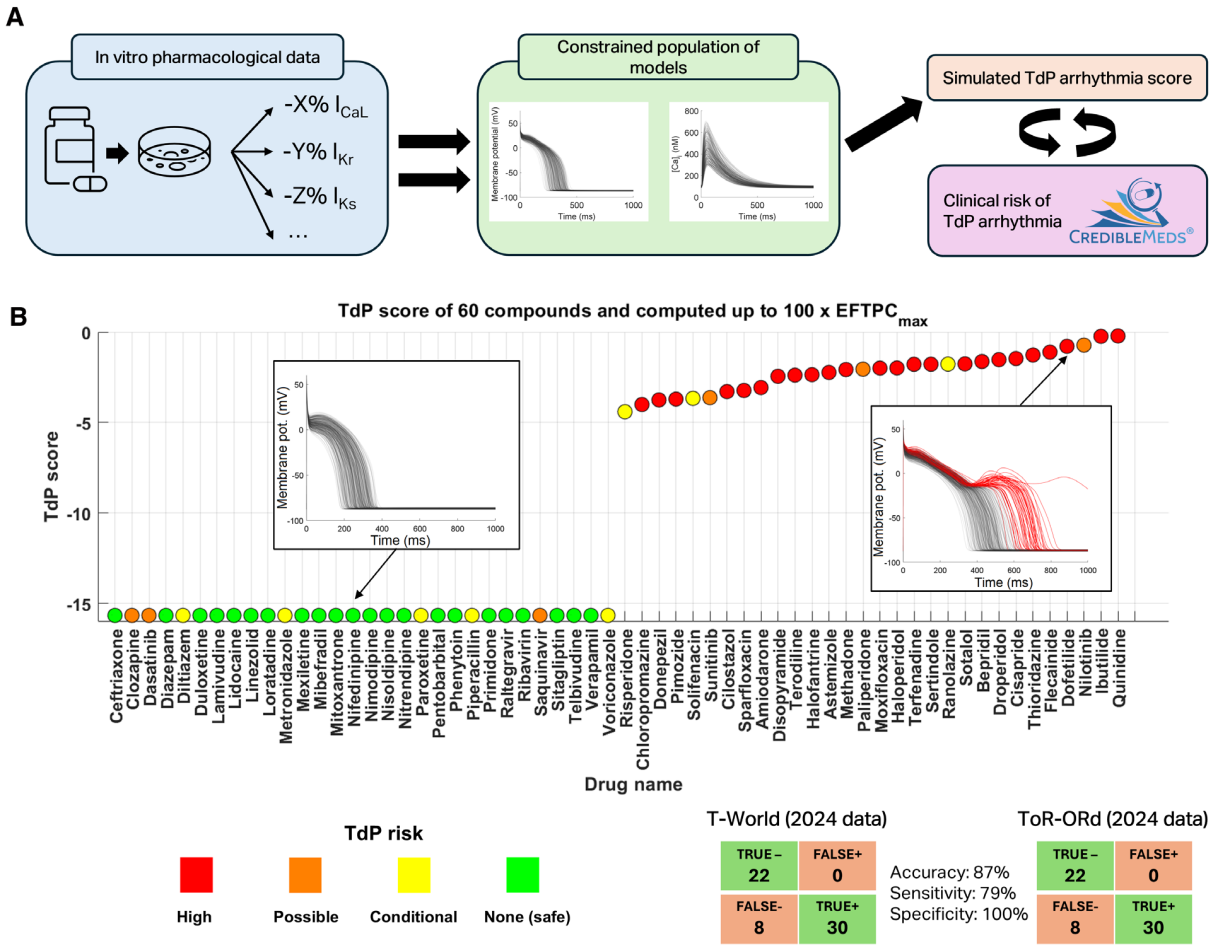
**In silico drug safety and efficacy assessment. A**, Schematic of the in silico drug trial procedure, showing how pharmacological data on dose-dependent inhibition of various currents by cardioactive drugs are applied to calibrated populations of models. Subsequently, arrhythmogenic behaviors (early afterdepolarizations [EADs] and repolarization failure) are detected and scored, and the prediction can be compared with reference clinical risk. **B**, Predicted risk scores for 60 drugs with T-World, with color-coded clinical risk, as in the study by Passini et al^10^ and Tomek et al.^25^ Tables of true/false classifications are provided in the right part for T-World and ToR-ORd. Please see the Methods section for a summary of how several data updates led to a subtly different performance of ToR-ORd in our study compared with the original publication.^25^ TdP indicates Torsades de Pointes.

Prediction of drug safety through in silico trials is a successful translational application of mechanistic computer models, with considerable uptake by industry and regulators.^10^ This is crucial as cardiac side effects are a major cause of drug attrition and market withdrawal.^23^ To demonstrate the utility of T-World for predicting drug-induced Torsades de Pointes, the most common cause of drug-induced proarrhythmia,^24^ we conducted an in silico trial using populations-of-models,^10,25^ comparing predictions against clinical risk data for 60 drugs (Figure 2A). Compared with prior works,^10,25^ we first updated drug safety annotations based on the most recent version of the CredibleMeds classification^17^ and pharmacological data for several compounds (Supplemental Methods).

A population of 341 models, constrained by experimentally informed human reference ranges for action potential duration (APD), calcium transient, and contraction biomarkers (Figure S2), was exposed to 60 drugs at doses up to 100× therapeutic levels. A previously established Torsades de Pointes risk score was calculated based on the number of models showing repolarization abnormalities at each concentration, inversely weighted by the drug concentration so that strongly supraphysiological concentrations yield a lower weight.^10^ Repolarization abnormalities comprise early afterdepolarizations (EADs) or repolarization failure. The model correctly predicted all no-risk drugs as safe with regard to Torsades de Pointes and all high-risk drugs as unsafe. Classifying the drugs into 2 categories of safe (no risk) and unsafe (high, possible, or conditional risk) yielded a prediction accuracy of 87%, with 79% sensitivity and 100% specificity (Figure 2B). This matches the prior state-of-the-art ToR-ORd model, highlighting the robustness of T-World despite its entirely different calcium-handling system, excitation-contraction coupling, and heavily revised ion current formulations.

**Figure 3.**
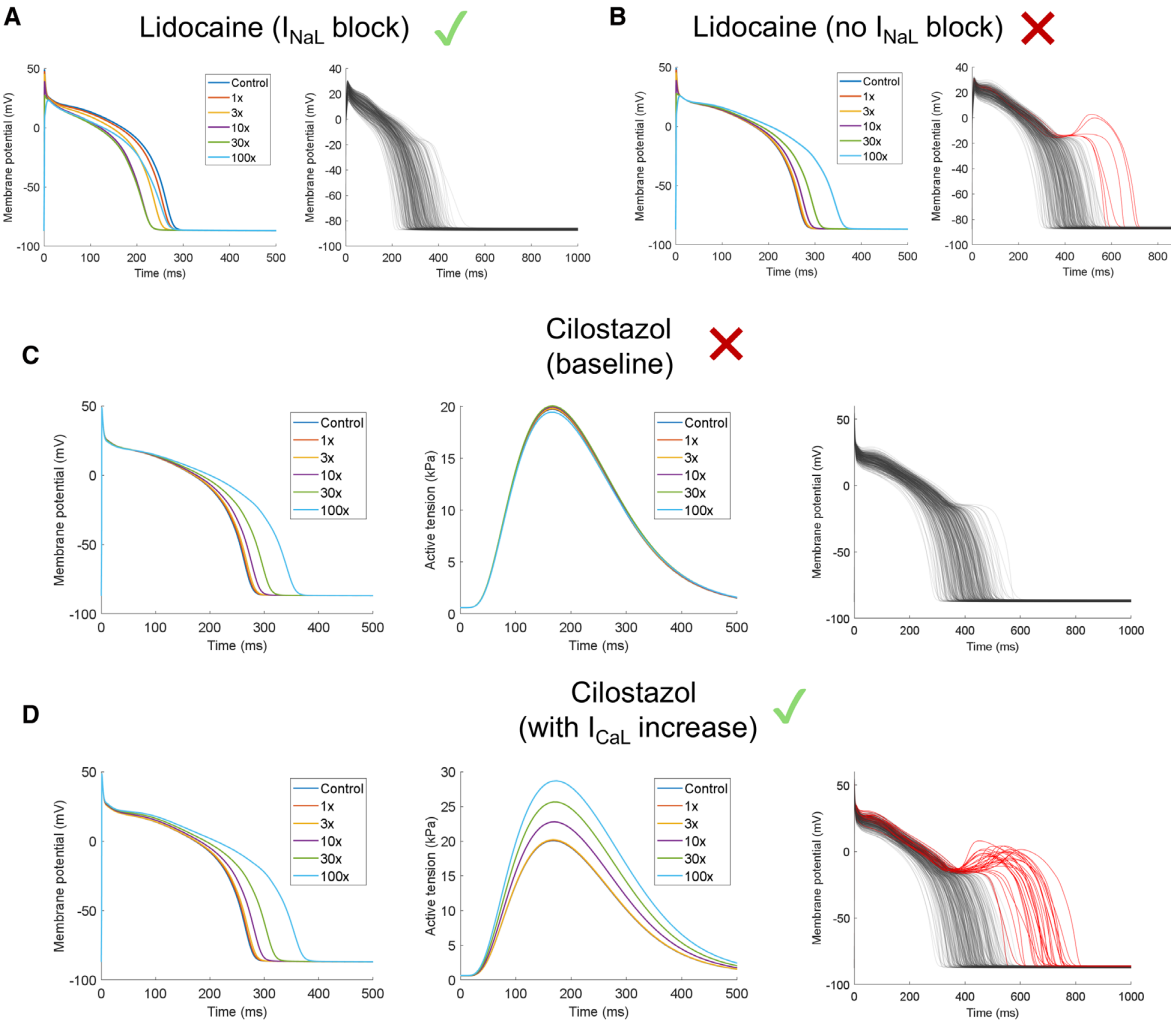
**Detection of problematic drug description data based on phenotypic disagreement. A**, Concentration-dependent effect of the first lidocaine description (with I_NaL_ effect) on the default T-world action potential (AP; **left**) and the effect of 100× on the population of models (**right**), showing overall safety (no model in the population manifests an early afterdepolarization [EAD]). **B**, Similar to **A** for the second lidocaine description available in the database (no I_NaL_ block), showing gradual dose-dependent AP duration (APD) prolongation to the left and repolarization abnormalities to the right. **C**, Simulated concentration-dependent effect of cilostazol as described by Kramer et al^27^ (IC_50_ for I_Kr_=13.8 µmol/L; IC_50_ for I_CaL_=91.2 µmol/L) on AP (**left**) and contractility (**middle**), showing AP prolongation, but no marked increase in contractility, and an absence of EADs with 100× in the population of models (**right**). **D**, Similar to **C** but including cilostazol-induced increases in I_CaL_ based on the study by Matsui et al^28^ (+10% at 10× dose=1.28 µmol/L, +22% at 30× dose, and +40% at 100× dose). This recapitulates the positive inotropic effect of the drug, as well as the proarrhythmic risk. I_CaL_ indicates L-type calcium current; I_Kr_, rapid delayed-rectifier potassium current; and I_NaL_, late sodium current.

A drug effect prediction can be reliable only when the underlying drug description data are accurate. We identified a new capability of T-World to detect incorrect pharmacological descriptions of drugs, which can limit prediction accuracy. When a drug with a known phenotypic effect (eg, changes to APD or contractility) is simulated, a discrepancy between the simulation and known reality indicates that an important effect of the drug is not included in the drug description data. We first illustrate this using lidocaine, a safe sodium channel blocker, where 1 of 2 available descriptions was excluded a priori during data curation. Both versions block peak I_Na_ and weakly block I_Kr_, with one additionally potently blocking I_NaL_. Lidocaine is known to shorten APD,^26^ and this is recapitulated only by the version including the I_NaL_ block, indicating its superiority (Figure 3A and 3B). Interestingly, the incorrect description generates EADs and falsely indicates a high arrhythmic risk (Figure 3A and 3B), highlighting the need to exclude incomplete drug descriptions. In this case, exclusion of the non-I_NaL_ formulation is independently supported by studies directly demonstrating I_NaL_ inhibition by lidocaine.^26^

Similarly, we identified an inaccuracy in the description of cilostazol, a high-risk PDE3 (phosphodiesterase 3) inhibitor with positive inotropic effects. The formulation by Kramer et al,^27^ historically used in in silico trials, characterizes cilostazol solely as a preferential I_Kr_ blocker with weaker secondary I_CaL_ inhibition. Simulating this formulation produces dose-dependent QT prolongation but virtually no change in predicted contractility (Figure 3C, left and middle), contradicting experimental data showing a marked increase in contractility after cilostazol exposure.^28^ When we incorporated PDE3 inhibition–mediated I_CaL_ enhancement via protein kinase A signaling (based on the study by Matsui et al^28^: +10% at 10× dose=1.28 µmol/L, +22% at 30×, and +40% at 100×), a clear procontractile effect emerged (Figure 3D, middle). At the highest dose (100×=12.8 µmol/L), peak active tension rose by 43% versus untreated cells, closely matching guinea pig data, showing a 35% increase with 10-µmol/L cilostazol. The PDE3 inhibition–driven I_CaL_ increase also markedly elevated arrhythmic risk, with many more models developing EADs than under the baseline formulation (Figure 3D versus 3C, right). Notably, APD prolongation was only slightly greater in the PDE3-inhibition model, indicating that APD alone cannot reveal the underlying issue in the baseline formulation. This underscores the importance of integrating multiple phenotypic markers when identifying gaps in pharmacological drug descriptions.

Simulation-based mechanistic models offer uniquely high-throughput methods for predicting how combinations of distinct channel-blocking drugs modulate human electrophysiology. As such, T-World can be used for studies on drug efficacy by identifying beneficial combinations of single-channel blockers or disentangling competing proarrhythmic and antiarrhythmic effects of multichannel drugs. For example, the multichannel blocker mexiletine was recently shown to be protective in a combined clinical and experimental study on long QT syndrome type 2,^29^ which arises from loss-of-function mutations in I_Kr_, which prolong APD. To explore the mechanistic basis of this protection, we simulated T-World with long QT syndrome type 2–like remodeling (70% I_Kr_ reduction and 82% I_NaL_ increase as in the study by Crotti et al^29^) and applied 2 published models of mexiletine action: MEX_Crumb_^30^ (also used in the drug safety study above) and MEX_Johannesen_^26^ (see Supplemental Methods for details). Both models primarily block I_NaL_ and I_CaL_ and partially I_Kr_, but only MEX_Crumb_ includes a substantial I_Ks_ inhibition.

**Figure 4.**
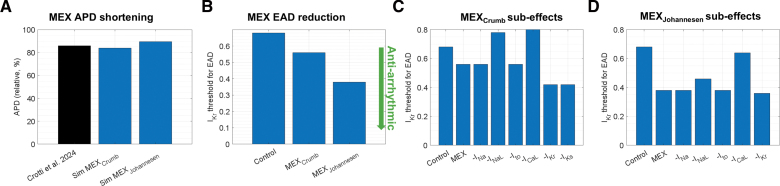
**Effects of mexiletine (MEX) on action potential duration (APD) and early afterdepolarization (EAD) vulnerability. A**, APD shortening by 2 distinct simulated MEX formulations (Supplemental Methods) compared with data in the study by Crotti et al.^29^ **B**, Reduction in EAD risk for both MEX formulations (quantified using an EAD threshold: the largest I_Kr_ multiplier sufficient to trigger EAD in a long QT syndrome type 2 [LQTS2] version of T-World at 0.25 Hz pacing). **C** and **D**, Comparison of EAD thresholds in the 2 MEX formulations, comparing an untreated LQTS2 cell, a cell treated with the simulated MEX formulation, and then a range of MEX treatments where the effects on individual ionic currents were turned off. An increase in those knockout bars compared with the full MEX effect indicates a protective effect of the given channel-blocking effect (omitting it makes EADs appear at a higher I_Kr_ level).

Both mexiletine variants shortened the APD and reduced EAD risk, consistent with clinical and experimental observations (Figure 4A and 4B). Interestingly, MEX_Johannesen_, which lacks the I_Ks_ block, was more potent against EADs despite inducing less APD shortening than MEX_Crumb_. To dissect these effects, we performed single-factor knockouts, a capability unique to computational models, by selectively turning off each ionic current effect of mexiletine in silico. For MEX_Crumb_, I_NaL_ and I_CaL_ blocks dominated the antiarrhythmic action, whereas I_Kr_ and I_Ks_ blocks were proarrhythmic in isolation (Figure 4C). For MEX_Johannesen_, I_CaL_ block and, to a lesser extent, I_NaL_ block were protective, while I_Kr_ and I_Ks_ effects were negligible or absent (Figure 4D). Given that there is no I_Ks_ block in this mexiletine model, and only a marginal I_Kr_ block at the given concentration, their effect on arrhythmic risk is none and small, respectively.

These findings suggest that not only I_NaL_ but also I_CaL_ inhibition contributes substantially to mexiletine’s antiarrhythmic effect. Further studies are needed to confirm or refute the drug’s effect on I_Ks_, as an I_Ks_-blocking drug could become risky in the setting of high sympathetic drive, where this current is responsible for maintaining repolarization reserve and offsetting I_CaL_ increase.

### Assessing Arrhythmogenesis in T2D

The generality of T-World enables the creation of predictive disease-specific models, capturing multiple aspects of the diseased phenotype. T2D is a major 21st-century epidemic linked to increased mortality, with cardiovascular disease as the leading cause of death. Sudden cardiac death from ventricular arrhythmia is the primary driver, yet the mechanisms behind ventricular arrhythmogenesis in T2D remain poorly understood.^31^ Limited human data on ionic currents and calcium-handling proteins^18^ show only partial alignment with heterogeneous animal studies. Progressive cardiac remodeling in T2D further complicates consistent disease characterization.

**Figure 5.**
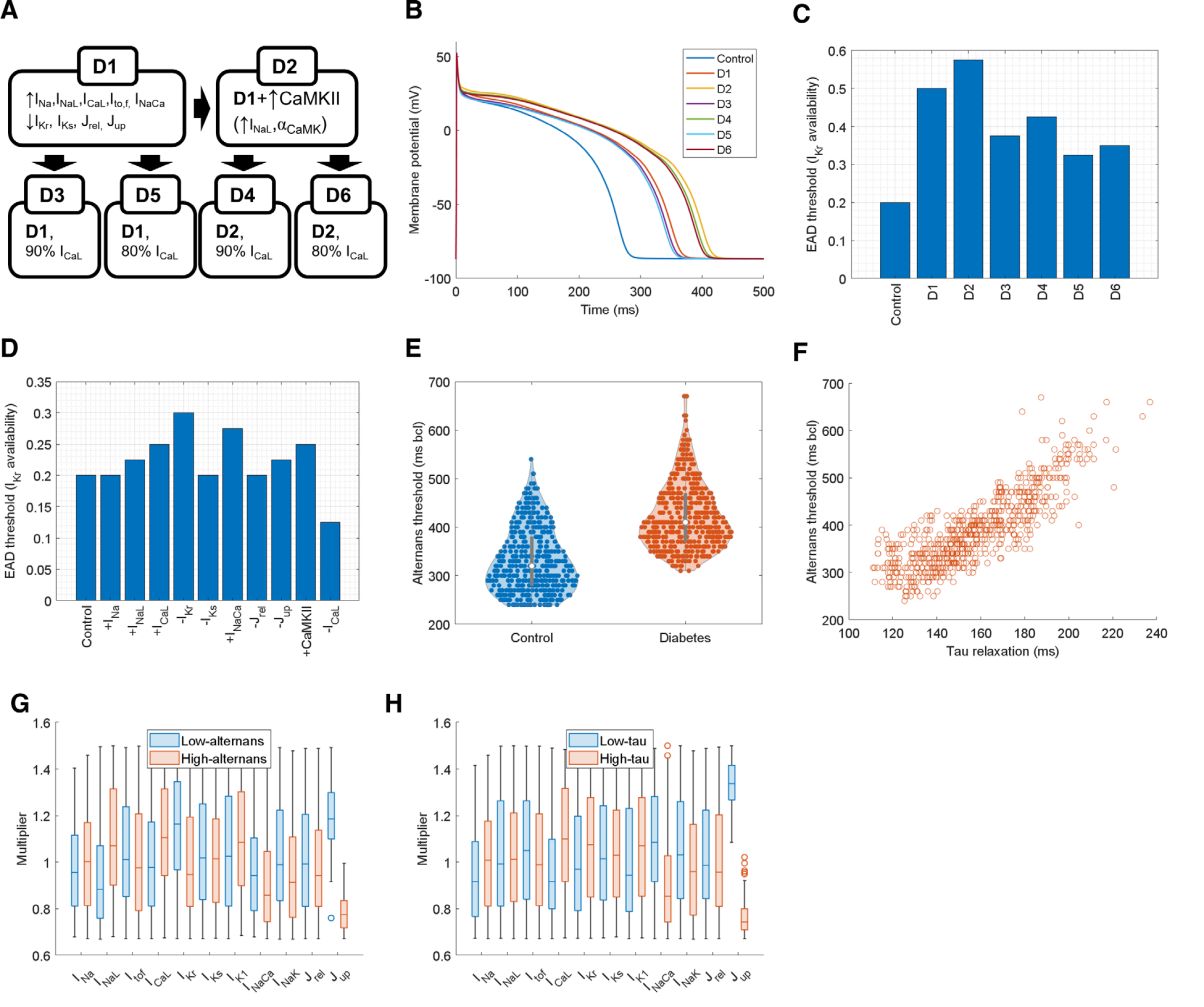
**Arrhythmogenesis promotion by type 2 diabetes (T2D). A**, An overview of 6 different versions of a diabetic model, reflecting heterogeneity of literature on T2D remodeling. D1 is the current best-estimate model of ionic remodeling in T2D, D2 represents additional Ca^2+^/calmodulin-dependent protein kinase II (CaMKII) activity, and D3 to D6 are derived models representing a reduction, rather than an increase in I_CaL_ (see Supplemental Methods for details and supporting literature). **B**, Comparison of steady-state action potentials of models, D1 to D6. **C**, Threshold for early afterdepolarizations (EADs) across control and diabetic models (the highest I_Kr_ availability, which supports EAD formation at 0.25 Hz pacing). Higher values indicate greater vulnerability to EADs. **D**, Similar to **C** when single elements of T2D remodeling considered in D1 to D6 are added to the control model. The +CaMKII column also involves an increase in I_NaL_, as described in the Methods section, and −I_CaL_ corresponds to 80% I_CaL_ density compared with the control model. **E**, Alternans threshold (the longest pacing cycle length, which induces calcium-transient alternans) between the population of control vs T2D models. The calibrated population of 786 models used in the Results section: stability of arrhythmic behaviors of the companion article^6^ was used as the control population, with T2D models created by adding diabetic remodeling from model variant D1 to each of those models. **F**, A scatterplot of tau of mechanical relaxation vs alternans threshold in the simulated T2D population from **E**. **G**, Distribution of parameter scaling constants in lower vs upper quartiles with regard to alternans threshold in the T2D population of models. **H**, Similar comparison of distributions, comparing lower vs upper quartile with regard to the tau of relaxation (high tau corresponds to poor relaxation). In both cases, SERCA (sarco/endoplasmic reticulum Ca^2+^ ATPase) pump availability (J_up_) is, by far, the clearest differentiating factor.

A significant portion of sudden cardiac deaths in T2D occurs in patients using potentially proarrhythmic drugs.^31^ This suggests hidden cardiotoxicity in T2D, with usually safe drugs (or safe drug concentrations) becoming dangerous. To investigate this, we created a range of models (D1–D6) reflecting key T2D phenotypes from the literature (Figure 5A), primarily using human data supplemented by animal studies (Supplemental Methods). All models exhibited action potential prolongation (Figure 5B), consistent with clinical QT prolongation in T2D.^32–34^ All diabetic model variants are more vulnerable to EADs (Figure 5C), manifesting EADs for greater availability (less inhibition) of I_Kr_. This includes T2D model variants with reduced I_CaL_ (D3–D6), which could be thought to be more protected given the central role of I_CaL_ in EAD formation. Key drivers of EAD risk were I_Kr_ reduction and I_NaCa_ (NCX-carried current) increase, further heightened by CaMKII hyperactivity and increased I_CaL_ in D1 and D2 (Figure 5D). I_CaL_ reduction alone (a component of D3–D6) showed reduced risk but not enough to offset other remodeling effects. This suggests that across different formulations of T2D remodeling, patients with T2D require less I_Kr_ inhibition to manifest EADs, therefore facing higher arrhythmia risk at drug doses considered safe for nondiabetic individuals.

A different arrhythmogenic behavior that is markedly increased in patients with T2D is alternans.^35^ We used the D1 version of T-World, which has recapitulated the clinical observation, showing alternans at slower pacing compared with nondiabetic versions (Figure 5E). Bonapace et al,^36^ furthermore, observed that alternans vulnerability is positively associated with diastolic dysfunction in patients with T2D. To investigate this phenomenon in T-World, we correlated the slowest pacing rate for calcium-transient alternans with diastolic function (tau of relaxation) in a population of models with perturbed parameters. Models prone to alternans exhibited impaired relaxation, consistent with clinical data (Figure 5F). We hypothesized and subsequently confirmed using T-World that reduced SERCA (sarco/endoplasmic reticulum Ca^2+^ ATPase) pump function can drive this relationship, given the association between SERCA availability and both relaxation and alternans vulnerability in simulated cardiomyocytes (Figure 5G and 5H).

### Na_V_1.8 Can Drive EADs in a Diseased Heart

**Figure 6.**
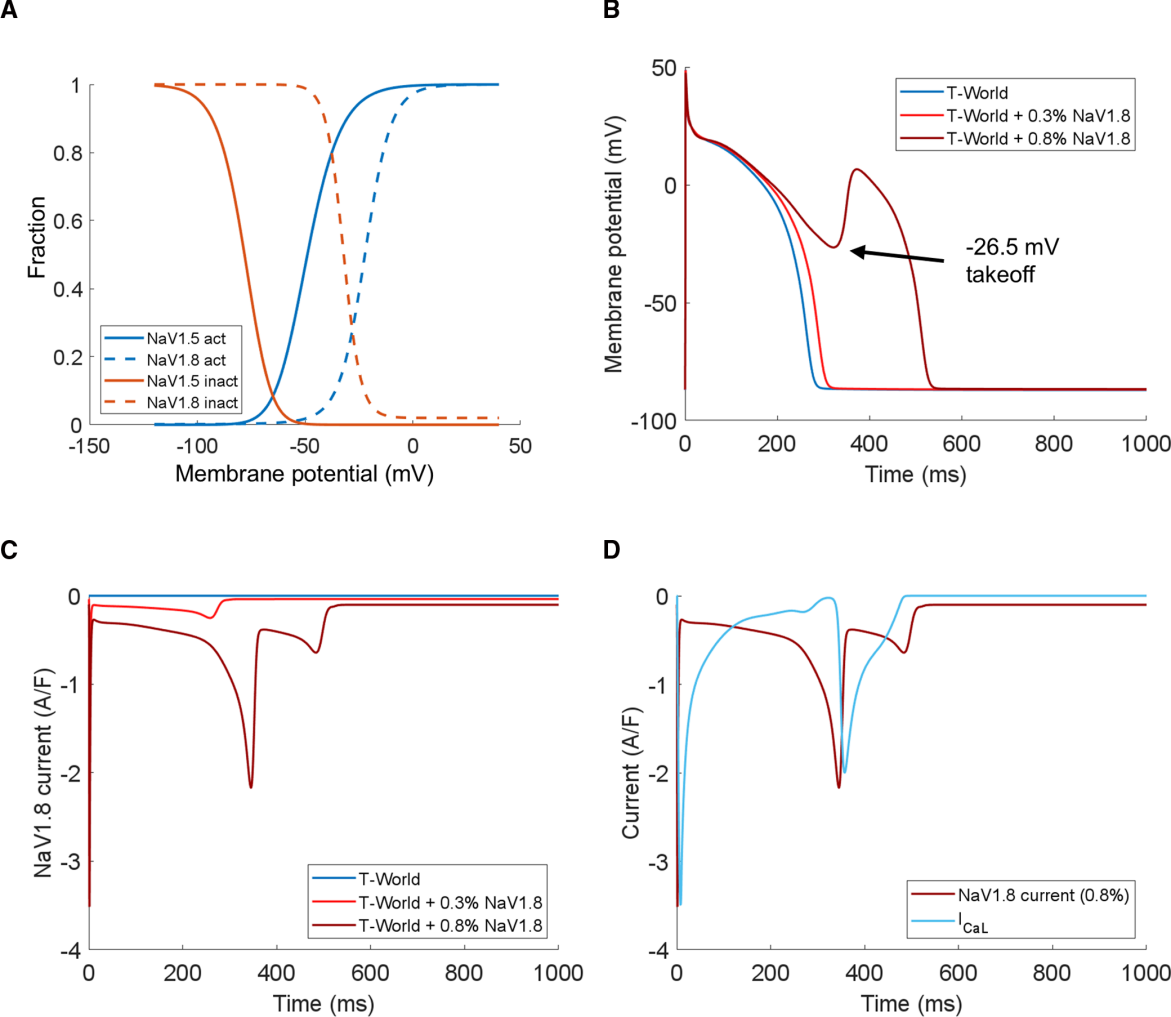
**NaV1.8 current as driver of early afterdepolarizations. A**, Activation and inactivation curves of the standard cardiac I_Na_ current through Na_V_1.5 vs right-shifted curves carried by the predominantly neuronal Na_V_1.8 (see Supplemental Methods for details).^40,41^ **B**, Action potentials (APs) of control T-World vs T-World during 1 Hz pacing with 2 different amounts of Na_V_1.8 current added (expressed as relative percentage of peak I_Na_). **C**, Na_V_1.8 current profiles during the APs in **B**. **D**, Concurrent visualization of Na_V_1.8 current and I_CaL_, demonstrating that the sodium current reactivation precedes I_CaL_.

Cardiac disease may remodel ionic currents active under physiological conditions, but it can also involve the expression of nonstandard ionic currents, absent in a healthy heart. We used T-World to investigate the role of Na_V_1.8, a primarily neuronal sodium channel subtype with recently much debated functionality in the heart. While Na_V_1.8 is minimally expressed in healthy hearts,^37^ it appears in hypertrophic or failing hearts and may contribute disproportionately to the late sodium current I_NaL_.^38,39^ Increased I_NaL_ can promote arrhythmias by prolonging APD (leading to EADs) or increasing sodium influx, reducing NCX calcium efflux, and causing delayed afterdepolarizations. However, given Na_V_1.8’s unique biophysical properties, including right-shifted activation and inactivation compared with Na_V_1.5 (Figure 6A), we hypothesized that it could directly generate EADs by providing depolarizing current during the late action potential plateau.

Introducing a small Na_V_1.8 current (≈0.3% of peak I_Na_) to T-World prolonged APD (Figure 6B), consistent with its role as an I_NaL_ source (Figure 6C). Strikingly, increasing Na_V_1.8 by 2.75-fold (to only 0.8% of peak I_Na_) was sufficient to trigger EADs at 1 Hz pacing (Figure 6B). These EADs emerged at a takeoff potential of −26.5 mV, clearly distinct from I_CaL_-driven EADs at −13 mV. Simultaneous tracking of the Na_V_1.8 current and I_CaL_ during EADs revealed that Na_V_1.8 initiates depolarization, subsequently activating I_CaL_ in a dual-current process (Figure 6D). Therefore, Na_V_1.8 can directly trigger EADs rather than merely prolong APD to facilitate I_CaL_ reactivation, potentially contributing to elevated arrhythmic risk in those patients.^42^ This mechanism suggests Na_V_1.8 as a possible antiarrhythmic target, for example, providing additional rationale for the use of ranolazine, which is protective in the hypertrophied heart^43,44^ and blocks Na_V_1.8.^45^

## Discussion

The newly developed human ventricular cardiomyocyte model, T-World, provides a mechanistically detailed and extensively validated framework for studying cardiac electrophysiology and excitation-contraction coupling across scales. In this study, we demonstrated its versatility in 4 domains: (1) linking cellular mechanisms to organ-level electrical activation, arrhythmia, and mechanical contraction; (2) assessing drug safety and efficacy; (3) elucidating causes of elevated arrhythmic burden in disease, exemplified by T2D; and (4) identifying the potential proarrhythmic consequences of noncanonical ion channel expression.

The vision of patient-specific organ-level models (digital twins) for personalized treatment of cardiac arrhythmias is drawing increasingly near although numerous challenges remain for implementation in clinical practice.^46,47^ Advances in noninvasive imaging, computational tools, and computing hardware have enabled the creation of sophisticated patient-specific models.^14^ Several proof-of-concept studies have shown the promise of these digital twins for improving, for example, risk stratification for sudden cardiac arrest and identifying targets for catheter ablation, and initial randomized clinical trials comparing usual care and digital twin-guided care have been published.^48–50^ However, to date, these studies have primarily focused on the structural substrate for cardiac arrhythmias, informed by magnetic resonance imaging and computed tomography, in part because of the challenges associated with organ-level simulations based on detailed single-cell models. We show that a biventricular organ-level model integrating the T-World cellular model reproduces human-like ECG morphology, ejection fraction, and PV loops, and can generate ventricular arrhythmia under physiologically relevant conditions without parameter tuning. These results position T-World as a powerful tool for multiscale investigations of cardiac arrhythmogenesis. Further research can also consider the suitability of the T-World for simulations of scars and ionic remodeling caused by myocardial infarction, similar to prior models,^13,51,52^ as well as coupling to fibrotic tissue,^53^ which alter conduction properties and arrhythmogenesis.^53–55^

We demonstrate T-World’s usefulness in preclinical drug safety testing, one of the most established translational applications of computational nonanimal methods, with significant industry adoption. T-World’s excellent performance in testing drug safety with regard to EAD-driven Torsades de Pointes arrhythmia through population-of-models in silico trials matches the prior state-of-the-art ToR-ORd^10,25,56^ while also enabling investigation of other mechanisms of arrhythmogenesis, including delayed afterdepolarizations, alternans, and steep restitution.^6^ We think that the barriers to further progress in drug safety prediction have now shifted from model fidelity to the completeness and reliability of experimental data describing compound actions, as shown by our data curation process and previously.^11^ Notably, we introduce a novel use of T-World simulations to identify inconsistencies in pharmacological data sets by employing its strong predictive performance of drug effects to test whether reported channel-blocking profiles reproduce known drug-induced phenotypes. Discrepancies between simulated and observed effects can reveal missing mechanisms in the drug description, guiding additional experimental measurements. This functionality is enabled by the mechanistic nature of T-World with functionally coupled outputs, such as arrhythmia risk, APD, or contraction, as opposed to data-driven, single-output predictor tools.

Missing effects likely contribute to the misclassification of several drugs in our study, which were labeled as safe by T-World, whereas, in reality, they have possible or conditional risk based on CredibleMeds.^17^ For example, clozapine is known to sometimes cause myocarditis and cardiomyopathy^57^ and may lead to sympathetic hyperactivity,^58^ neither of which is captured by the channel-blocking description of the drug. Voriconazole appears to interact with the metabolism of other, possibly riskier drugs,^59^ which could explain its conditional proarrhythmic effect in patients. The data on the action of voriconazole used in our study indicate that it blocks I_CaL_ and I_Kr_ (the former slightly more), which would be a generally safe profile, if these were the sole effects. Dasatinib is a tyrosine kinase inhibitor used in cancer therapy, which targets multiple signaling pathways and may promote heart failure in some subjects,^60^ so it is also possible that it acts beyond direct QT prolongation via channel block. Furthermore, it should be noted that acute drug effects typically assessed in preclinical proarrhythmia screening can differ from chronic effects due to drug-induced modulation of signaling cascades involved in long-term regulation of ion channel expression and trafficking. For example, the distinct proarrhythmic risk of different I_Kr_ blockers correlates with their long-term potentiation of I_NaL_ via phosphoinositide 3-kinase/Akt signaling.^61^ T-World is optimally positioned to assess the proarrhythmic risk of these acute and chronic effects.

There are multiple directions in which T-World may be applied in the future in pharmaceutical research and industry. The first is to focus on drug efficacy. Here, we used T-World to understand the antiarrhythmic potency of mexiletine in long QT syndrome type 2. However, in addition to understanding multichannel blockers in this way, T-World will be useful for finding combinations of channel-blocking drugs, which offer maximum therapeutic benefit. For example, the combined application of Na^+^- and K^+^-channel-blocking drugs has been shown to increase antiarrhythmic efficacy in animal models.^62^ Second, with T-World’s representation of sex differences and the suitability for modeling disease, studies on drug safety and efficacy will be increasingly possible in a sex-^63^ and disease-specific manner. Finally, with its representation of contractility and improved excitation-contraction coupling, T-World is well-suited for studying drug effects on contractility, a rapidly developing domain of applications with high relevance for industry.^9,11^ An advantage of highly general tools such as T-World as opposed to single-purpose predictors is that they enable compound queries, such as find drugs reducing arrhythmic risk, without compromising contractility. At the same time, the proposed future trends will require additional layers of validation and will benefit from integration with pharmacokinetics and pharmacodynamics simulations.^64^

Arrhythmias pose a significant risk in heart disease, and T-World’s generality makes it ideal for studying complex cardiac diseases. Using T-World, we constructed a pilot cell model for T2D, a condition with high arrhythmic burden but limited mechanistic understanding.^31^ The model revealed increased risks of EADs and alternans, which can explain elevated rates of sudden cardiac death in this population. High alternans vulnerability was associated with diastolic dysfunction in T-World simulations, matching clinical data.^36^ Furthermore, the higher EAD risk indicates a heightened vulnerability to drug-induced arrhythmia, a major concern in T2D.^31^ Despite promising results achieved, we note the urgent need to collect new, high-quality human data sets to characterize and understand how T2D dysregulates the heart, given the paucity of existing data.

T-World can be applied to study the role of nonstandard channels present only in disease. Here, we investigated the role of the brain-type Na_V_1.8 channel, which is absent in healthy hearts,^37^ but can appear in disease, for example, in heart failure.^38,39^ We show that even if present in small amounts, Na_V_1.8 may directly contribute to arrhythmia through its unusual activation and inactivation properties, highlighting it as a potential treatment target. More sophisticated models of cardiac Na_V_1.8 current may be developed in the future, for example, taking into account the fact that Na_V_1.8 is modulated by CaMKII,^39^ and the channels may be, thus, more active at faster heart rates.

In summary, T-World represents a broadly applicable, human-specific computational framework that bridges cellular-scale mechanisms with organ-level and translational outcomes. Its mechanistic fidelity, versatility, and open-source availability position it to accelerate progress in cardiovascular research, pharmaceutical development, and precision medicine. By integrating across scales and domains, T-World exemplifies how next-generation in silico models can enhance mechanistic understanding, reduce reliance on animal testing, and inform the design of safer and more effective cardiac therapies.

## ARTICLE INFORMATION

### Acknowledgments

The authors thank Eleonora Grandi, Stefano Morotti, and Haibo Ni for useful discussions on how models derived from Shannon et al operate. The authors also thank Dirk Gillespie, Dezso Boda, Pavel Jungwirth, and Geir Halnes for their insights on how ionic driving force through open L-type calcium channels should or should not be modeled. The authors also thank Roshni Shetty and David Ortega for spotting minor issues in the code and Marketa Tomkova for proofreading the text.

### Author Contributions

J. Tomek conceptualized and coordinated the study and performed cell-level simulations. J. Heijman, B. Rodriguez, and D.M. Bers jointly supervised the project. B. Rodriguez, A. Bueno-Orovio, and A.I. Hasaballa acquired funding for organ-scale simulations. M. Holmes, A.I. Hasaballa, and Z.J. Wang performed 3-dimensional contraction modeling. H. Martinez-Navarro and L. Arantes Berg performed a 3-dimensional simulation of electrophysiology and arrhythmia. X. Zhou supported the drug safety assessment. A. Bertrand contributed to the design of the type 2 diabetes model. J. Tomek and J. Heijman wrote the initial draft, subsequently revised by B. Rodriguez, D.M. Bers, A. Bertrand, M.A. Colman, and A. Bueno-Orovio.

### Disclosures

None.

### Supplemental Material

Supplemental Methods

Table S1–S3

Figures S1–S2

Videos S1–S2

Major Resources Table

References 51,65–100

## Supplementary Material


