# Supplementary material for: T-World Virtual Human Cardiomyocyte. II. Organ-Scale Simulations and Applications
Source: Circ Res. 2026 Apr 8;138(10):e328123. doi: 10.1161/CIRCRESAHA.125.328123 (PMC13152080; doi:10.1161/CIRCRESAHA.125.328123)
Supplement: Supplementary file 3 [file res-138-e328123-s003.pdf]

Supplementary Methods

Organ-level biventricular simulations of electrophysiology

Simulations were conducted using a multiscale computational framework for simulating human cardiac electrophysiology from ionic currents to electrocardiographic signals under healthy and ischemic conditions using the GPU-based solver MonoAlg3D[65] and as described in [12,13]. The aim was to analyse the behaviour of the cellular model to trigger and sustain ventricular arrhythmias when implemented in biventricular meshes obtained from clinical MRI data. As described in [12,13], the monodomain model was used over a human biventricular model, calibrated and evaluated using extensive clinical and experimental data, both under healthy and ischemic conditions, and at multiple scales including cell (ion dynamics, action potential), tissue (conduction velocity, fibre anisotropy), and organ (activation sequence, electrocardiographic signal, electrophysiological heterogeneities) levels. Fibre orientation in the myocardium was represented using the rule-based method by Doste et al.[66] reproducing experimental findings[67].

Given the high pro-arhythmic potential of early (phase 1A) myocardial ischemia[68], we simulated acute regional ischemia in the anterior myocardial wall caused by left-anterior descending (LAD) artery occlusion to assess the cellular model's capabilities to trigger and yield pro-arrhythmic behaviours at an organ scale as in [12]. Electrophysiological alterations derived from acute ischaemia, caused by hyperkalaemia, hypoxia, and acidosis, were added into the T-World model in ischaemic area to model the changes in refractoriness and conduction velocity in humans[20,69]. The formulation of the ATP-dependant potassium current $IK_{(ATP)}$ [70] was incorporated to simulate hypoxia. Electrophysiological alterations in the ischaemic zone were included considering the ischaemic core zone (ICZ) and the lateral border zone (BZ), which represent an electrophysiological gradient between the ICZ and the remote myocardium[20]. The subendocardial border zone was included to account for the oxygen diffusion from blood in the ventricular cavities, reported experimentally[71].

Sinus rhythm was simulated to obtain realistic ECGs, implemented through a realistic patient-specific model of the ventricular conduction system based on previous work[13,14]. Cardiac activity at rest was simulated by sinus rhythm pacing at basic cycle length (bcl) of 800 ms. As described in [12,13], arrhythmia was induced by a stimulation protocol with progressively shorter cycle length (2 beats at bcl of 400 ms, 2 beats at 350 ms bcl, 4 beats at 300 ms, 6 at 250 ms bcl) taking approximately 4 seconds to complete the pacing protocol. No further pacing was induced until the end of the 10-second simulation to assess arrhythmia sustenance.

Simulation of 9 seconds took approx. 8 days on a single PC with a Titan RTX GPU. The simulation time is likely to be much faster on a computer with a professional-grade GPU suitable for double precision calculations.

Organ-level biventricular simulations of electrophysiology and contraction

Electromechanical simulations were performed using our multi-scale framework described in previous works with extensive calibration and validation.[16,63,72] A biventricular anatomical mesh was generated from previously published CT image data.[73] Electrical propagation was simulated using the monodomain equation incorporating orthotropic diffusion along the fibre, sheet and sheet-normal directions[66] and the novel T-World model incorporating realistic QRS morphology [74] and T-wave morphology using methods described by Camps et al.[75] For the Alya simulator used for mechanical contraction simulations, we translated the code manually into Fortran (and

then carried out side-to-side simulations comparing outputs to verify the process). Simulations were conducted using the finite element method with linear 4-node tetrahedral elements. The mesh consists of 212,404 nodes and 1,093,149 elements and its simulation takes approx. 5 hours per beat on a supercomputer with 10 CPU nodes (1280 cores).

Strongly-coupled electromechanics were modelled as in,[76] with orthotropic passive mechanical behaviour and linear momentum balanced with inertial effects. Unlike previous models, cellular active tension is scaled using a sigmoidal model, where the active tension in each node, $\mathrm{AT_{scaled}}$ is calculated as follows:

$$\mathrm{AT_{scaled}} = \mathrm{AT_{cell}} \times \left(\mathrm{d} + \frac{\mathrm{a-d}}{1+\left(\frac{\mathrm{AT_{cell}}}{\mathrm{c}}\right)^{\mathrm{b}}} \right), \ \mathrm{a} = 4, \mathrm{b} = 2, \mathrm{c} = 18, \mathrm{d} = 15$$

We employed this approach given that T-World shows non-negligible active tension during diastole. Presence of active tension during diastole is in line with experimental with evidence[77,78], and in contrast with prior ToR-ORd+Land[79], where diastolic tension is minimal. Hence, although scaling by a fixed constant maintained relatively low diastolic active tension in ToR-ORd+Land, it would produce excessively high diastolic active tension in T-World. For this reason, a sigmoid scaling function is employed, maintaining low active tension during diastole, but amplifying active tension during systole.

A five-phased state machine was added to capture the behaviors of diastolic inflation, and elastic recoil. Mechanical boundary conditions and passive mechanical inputs are as presented in Wang et al.[16] A detailed description of the cardiac baseline mechanical model is available in.[76,80] A brief summary of the five-phase state machine [16] is provided below:

Two independent piece-wise functions describe the five-phases of the cardiac cycle, to prescribe the values of pressures applied to the endocardial surface of the left and right ventricle, P_{LVendo} and P_{RVendo} respectively. From the beginning of the simulation, we describe the five phases chronologically.

1. *Active diastolic filling*: To mimic the effect of atrial contraction, the endocardial pressures are linearly increased over time t_{EDP} to reach the end-diastolic values.
2. *Isovolumetric contraction*: When electrical activation occurs, and active contraction begins to develop, the ventricular pressures are controlled such that volume is maintained constantly via the following equations:

$$dP_{LVendo} = -\frac{1}{C_{P_{LV}}} dV_{LVendo} - \frac{1}{C_{V_{LV}}} \frac{dV_{LVendo}}{dt}$$

$$dP_{RVendo} = -\frac{1}{C_{P_{RV}}} dV_{RVendo} - \frac{1}{C_{V_{RV}}} \frac{dV_{RVendo}}{dt}$$

   Where $C_{P_{LV}}, C_{P_{RV}}, C_{V_{LV}}, C_{V_{RV}}$ are inverse penalty terms for the volume difference and volume rates for each ventricle. These volume rate terms stabilize against artificial oscillations of ventricular pressure which may arise due to inertial effects.

3. *Ejection*: Once ventricular pressure exceeds aortic pressure (P_{AO}) and pulmonary artery pressure (P_{PA}), ejection begins. A two-element windkessel model is used to model the blood pressure of the systemic circulation system, where the compliance of the aortic and pulmonary arteries, C_{AO} and C_{PA} respectively, are described by

$$C_{AO}\frac{dP_{AO}}{dt}+\frac{P_{AO}}{R_{AO}}=\frac{-dV_{LVendo}}{dt}$$

$$C_{PA}\frac{dP_{PA}}{dt}+\frac{P_{PA}}{R_{PA}}=\frac{-dV_{RVendo}}{dt}$$

With R_{AO} and R_{PA} being the resistance of the aortic and pulmonary circuits. During this phase, the ventricular pressures are modelled as equal to the arterial pressures, disregarding negligible pressure gradients across the arterial valves.

$$dP_{LVendo}=\frac{-1}{C_{pLAV}}(V_{LVendo}-DSV_{LV})-\frac{1}{C_{vLAV}}\frac{dV_{LVendo}}{dt}$$

$$dP_{RVendo}=\frac{-1}{C_{pRAV}}(V_{RVendo}-DSV_{RV})-\frac{1}{C_{vRAV}}\frac{dV_{RVendo}}{dt}$$

Where $C_{pLAV}, C_{pRAV}, C_{vLAV}, C_{vRAV}$ were the inverse of the penalty terms for volume different to DSVs and volume rates for each ventricle.

4. *Isovolumetric relaxation*: Following the same formulation as for isovolumetric contraction, this phase is initiated when the ventricular flow reverses.
5. *Passive filling*: Once the endocardial pressure drops below the defined left and right atrial pressures, P_{LA} and P_{RA}, the pressure is prescribed so that the two ventricular volumes return to their diastasis value (DSV_{LV}, DSV_{RV}).

Throughout these phases, ventricular volumes V_{LVendo} and V_{RVendo} were calculated and updated using the divergence theorem, with the assumption that volume was constrained by a flat lid located on the aortic and pulmonary valvular planes for the right and left ventricles respectively.

Drug safety prediction using population of models

We generated a population of models for the drug safety assessment study, representing variation in ionic currents and calcium handling [10,25,56]. We generated a population of 1000 models, with the following randomly sampled perturbations to ionic currents and fluxes versus baseline: 50-200% (I_{Na}, I_{NaL}, $I_{to,f}$, I_{CaL}, I_{K1}), 25-125% (I_{Kr}, I_{Ks}), 50-150% (J_{rel}, J_{up}), and 75-175% (I_{NaCa}, I_{NaK}). The ranges were chosen a priori to enable sufficient phenotypic diversity including vulnerability to early afterdepolarizations (EADs). 341 out of the 1000 models passed all the calibration criteria based on comparing biomarkers of the simulated models to experimentally observed ranges in human [79,81,82], summarized in **Table S1** (also used as Table S6 in the supplement of the companion manuscript).

Table S1. Biomarker ranges for the calibration of population of models used to test drug torsadogenicity. *Based on studies summarized in [79,81]. APD = action potential duration. CaT = calcium transient.*

Biomarker	Minimum	Maximum
AP peak	7 mV	55 mV
Resting membrane potential	-95 mV	-80 mV
Peak upstroke velocity	100 V/s	1000 V/s
APD90 (Action potential duration at 90% recovery)	180 ms	440 ms
APD50 (APD at 50% recovery)	110 ms	350 ms
APD40 (APD at 40% recovery)	85 ms	320 ms
90-40 triangulation (APD90-APD40)	50 ms	150 ms
CaT duration at 90% recovery	220 ms	750 ms

CaT duration at 50% recovery	120 ms	420 ms
CaT amplitude	200 nM	600 nM
Peak of CaT	200 nM	1000 nM
Diastolic Ca	0 nM	400 nM
Peak active tension	5 kPa	40 kPa
Time to peak active tension	120 ms	200 ms
Time from peak active tension to 95% recovery	200 ms	600 ms

The population was used to test 60 reference compounds with known arrhythmic risk at multiple concentrations versus the therapeutic dose: 1x, 3x, 10x, 30x, and 100x, using drug IC_{50} values and Hill coefficients as in [10]. The occurrence of drug-induced repolarization abnormalities, such as EADs, in the population of models was assessed and was used to calculate the Torsades de pointes (TdP) score as in [10]. CredibleMeds[83] was used as gold standard for clinically established TdP risk, dividing the reference compounds in four categories: 1, high risk; 2, possible risk; 3, conditional risk; 4, safe (when not included in CredibleMeds). We used the most recent version of the CredibleMeds data, which differs from data used previously as the clinical reference [10,25], slightly reducing the performance of our model compared to other studies, as detailed in Results.

Compared to comparable prior *in silico* trials on this battery of compounds [10,25], which used 62 compounds, we excluded $BaCl_2$. This toxic salt is to our knowledge not used medically, and it is unlikely it would be listed in Crediblemeds in the first place. We did nevertheless confirm the drug is arrhythmogenic at higher concentrations when the population is exposed to it (not shown), which is in line with a range of experimental studies indicating its arrhythmogenic profile [84–86]. We also excluded procainamide, on the grounds that it is mainly its metabolite N-acetylprocainamide with different properties that causes arrhythmia, rather than the drug itself, for which IC50 values were obtained[87–91].

Based on experience when running studies drug safety prediction in prior models [10,25], we additionally curated the data describing channel blockade applied to the baseline model when we were aware of omission of an important drug effect in the data. This affected the formulation of 4 drugs: lidocaine, mexiletine, amiodarone, and cilostazol. For lidocaine and mexiletine, two near-identical formulations were present in the source data, differing only in their effect on late sodium current I_{NaL} (In silico trials simulate multiple drug formulations when multiple source datasets describing pharmacological effects are available, using the most hazardous prediction to generate the final arrhythmic score). Given that it is well established that these two drugs block I_{NaL} [26] (in fact, preferentially over I_{Na}), we decided to exclude the formulations without I_{NaL} inhibition. Following this, we confirmed that no other drugs in the dataset should be excluded because of two near-identical formulation, differing only in inclusion of I_{NaL} effect. In the case of amiodarone, potent chronic effects on key repolarizing potassium currents were reported [92,93]. In line with the study by Kamiya et al. [92], which used 100 mg/kg/day (ca. 10fold of therapeutic dose for arrhythmia treatment [94]), we applied the observed effect for simulations of 10x concentration and greater: a 29% and 83% reduction in I_{Kr} and I_{Ks}, respectively. For cilostazol, the described IC_{50} values describe direct channel block, but entirely ignore the primary effect of the drug, i.e., inhibition of PDE3, leading to increased I_{CaL}, the main ionic current promoting EADs and TdP. Consequently, we increased I_{CaL} by +10% at 10x concentration, +22% at 30x, and +40% at 100x, based on Figure 2 in [28] (100x of therapeutic dose is 12.8 μM).

We note that when comparing the performance of T-World to ToR-ORd, the latter's performance differs subtly from the source 2019 publication where in silico drug assessment was also carried out [25]. This comprises two changes: First, the annotations of diltiazem and piperacillin changed from *no risk* to *conditional* risk in the reference data, leading to two additional false

negatives in the current article, as both T-World and ToR-ORd predict the drugs to be safe. Second, a problematic formulation of lidocaine-induced changes was excluded in initial data curation carried out in this study, leaving a single other formulation of lidocaine in the dataset. Given that this formulation was driving a false positive result in our previous article from 2019 [25], we excluded this false positive from comparison with T-World for maximally fair comparison (otherwise T-World performance would be enhanced artificially).

Analysis of mexiletine efficacy in Long QT syndrome type 2

When simulating the effect of mexiletine in LQTS2, we explored two formulations of mexiletine: MEX_{Crumb} (the same formulation as used in the in silico trial above, based on the data by Crumb et al. [30]) and $MEX_{Johannesen}$, based on a different set of measurements [26]. In the data by Johannesen et al., I_{Na} and $I_{to,f}$ were not measured, and in the absence of measurements, we inferred the relative channel blocks based on the Crumb et al. IC50 values and Hill coefficients. Drug concentrations were chosen to produce an approximately data-like change in action potential duration [29]: 25 μM for MEX_{Crumb} and 10 μM for $MEX_{Johannesen}$. The effects on ionic currents at the given concentrations are summarized in (**Table S2**). LQTS2 was modelled as a 70% reduction in I_{Kr} combined with an 82% increase in I_{NaL} (the I_{NaL} increase was described by Crotti et al. [29]).

Table S2. Multipliers of ionic currents by two formulations of mexiletine. *Please note that the lack of effect of mexiletine on I_{Ks} in $MEX_{Johannesen}$ follows from a direct measurement showing no visible effect, it is not due to a mere lack of data.*

Ionic current	MEX_{Crumb} (25 μM)	$MEX_{Johannesen}$ (10 μM)
I_{Na}	0.656086250445016	0.818648051180129
I_{NaL}	0.192007760172465	0.5335
$I_{to,f}$	0.920173174700735	0.96368476372007
I_{CaL}	0.827890160668684	0.805
I_{Kr}	0.756451488675938	0.9124
I_{Ks}	0.560748793105810	1

Type 2 diabetes modelling

Data on cellular remodeling in Type 2 diabetes (T2D) in human remain rare, with animal data being heterogeneous and dependent on a specific animal model chosen [95]. We based our baseline model of T2D on the most comprehensive human-based dataset by Ashrafi et al. [18]. Compared to the model constructed there, we reduced the extent of I_{Kr} reduction and I_{NaCa} increase, as inclusion of the original values leads to unrealistically exaggerated phenotype (extremely long action potential and very small calcium transient). In addition, we omitted the change in I_{K1}, given relatively consistent data in animals suggesting no major change [95]. Given studies indicating reduced SERCA pump activity in diabetes, we have reduced SERCA pumps by 20% [96,97]. We label this baseline diabetic model D1 (**Table S3**).

To explore additional effects reported in literature, we formed model D2 by taking D1 as a starting point and doubling I_{NaL} and increasing activity of CaMKII by increasing the α_{CaMKII} parameter by 50% (**Table S3**), reflecting I_{NaL} and CaMKII increase described in the literature [98–100].

Finally, a part of animal-based studies on T2D indicates reduced, rather than slightly increased I_{CaL}. Given the importance of I_{CaL} for the risk of EAD formation and thus arrhythmogenesis, we explored this possibility by constructing D3, D5 (based on D1) and D4, D6 (based on D2) by setting I_{CaL} availability to 90% and 80% of I_{CaL} compared to undiseased T-World (**Table S3**). The reductions replace the I_{CaL} increase of D1 and D2; they are not used to merely multiply it.

Table S3: Overview of T2D model versions explored in the article. *Percent given represent relative change to baseline (i.e., it is not additional).*

Model	Description
**D1**	121% I_{Na} and I_{NaL}, 114% I_{CaL}, 126% $I_{to,f}$, 70% I_{Kr}, 95% I_{Ks}, 150% I_{NaCa}, 90% J_{rel}, 80% Jup (SERCA)
**D2**	D1, 200% I_{NaL}, 150% α_{CaMKII}
**D3**	D1, 90% I_{CaL}
**D4**	D2, 90% I_{CaL}
**D5**	D1, 80% I_{CaL}
**D6**	D2, 80% I_{CaL}

NaV1.8 current investigation

A separate version of T-World including the NaV1.8 current was created by adding the Choi-Waxman formulation [19] to a baseline model. The equations for this two-gate Hodgkin-Huxley model are reproduced below. The only change to the original version is a small update to h_∞, giving it a small degree of activity during action potential plateau, in line with its nature as a contributor to late sodium current.

$$\alpha_{m,NaV1.8} = 2.85 - \frac{2.839}{1 + e^{\frac{V-1.159}{13.95}}}$$

$$\beta_{m,NaV1.8} = \frac{7.602}{1 + e^{\frac{V+46.463}{8.8289}}}$$

$$m_{\infty,NaV1.8} = \frac{\alpha_{m,NaV1.8}}{\alpha_{m,NaV1.8} + \beta_{m,NaV1.8}}$$

$$\tau_{m,NaV1.8} = \frac{1}{\alpha_{m,NaV1.8} + \beta_{m,NaV1.8}}$$

$$h_{\infty,NaV1.8} = 0.02 + \frac{0.98}{1 + e^{\frac{V+32.2}{4}}}$$

$$\tau_{h,NaV1.8} = 1.218 + 42.043 \cdot e^{\frac{-(V+38.1)^2}{2 \cdot 15.19^2}}$$

$$\frac{dm_{NaV1.8}}{dt} = \frac{m_{\infty,NaV1.8} - m_{NaV1.8}}{\tau_{m,NaV1.8}}$$

$$\frac{dh_{NaV1.8}}{dt} = \frac{h_{\infty,NaV1.8} - h_{NaV1.8}}{\tau_{h,NaV1.8}}$$

The ionic current through NaV1.8 in a compartment X (dyadic or subsarcolemmal) is

$$I_{NaV1.8,X} = f_X \cdot g_{NaV1.8} \cdot m_{NaV1.8} \cdot h_{NaV1.8} \cdot \left(V - E_{Na,X}\right)$$

The variable f_X is the fraction of the current in compartment X. Like with most other currents, 11% of the current was placed in the dyadic compartment, with the rest placed in the subsarcolemmal one. $E_{Na,X}$ is the equilibrium potential for sodium in the given compartment.

For the purpose of simulations visualized in Figure 5 of the main manuscript, the three explored values of the conductance of $I_{NaV1.8}$ ($g_{NaV1.8}$) were 0, 0.1085, and 0.3 mS/μF (all substantially lower than the conductance of the main NaV1.5 current I_{Na} with g_{Na} > 22 mS/μF).

Please note that only the NaV1.5 current is used in T-World by default, with NaV1.8 an optional addition relevant in modelling, e.g., heart failure.

Supplementary figures

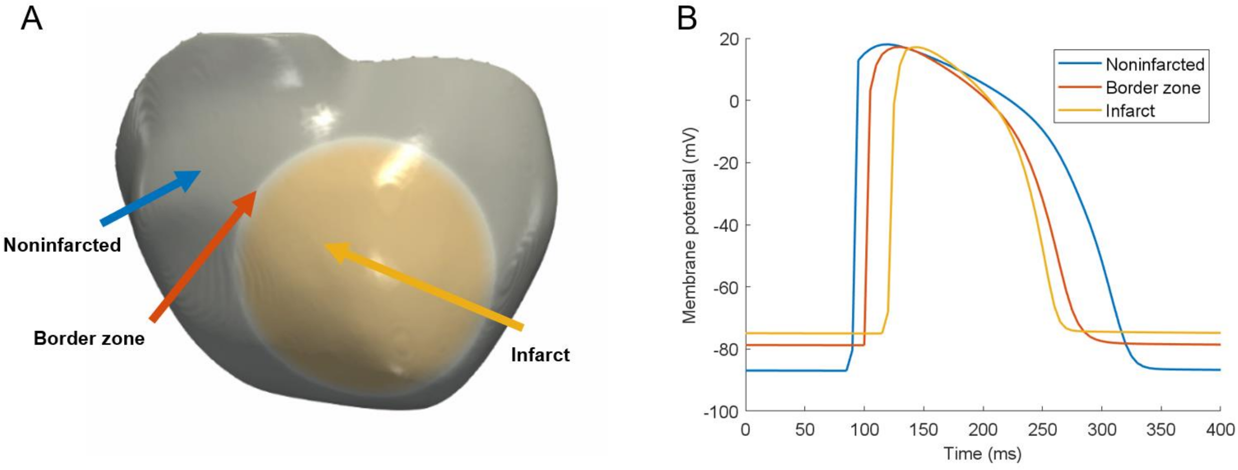

Figure S1. Action potentials in distinct heart regions. A) *Three distinct locations in a biventricular model used in Figure 1B in the main article.* ***B)*** *Corresponding action potentials (within the 820-1220 ms time interval of the simulation, regular pacing phase), showing depolarization and action potential shortening in the infarct zone. Membrane potential was stored at the temporal resolution of 1 sample per 5 ms.*

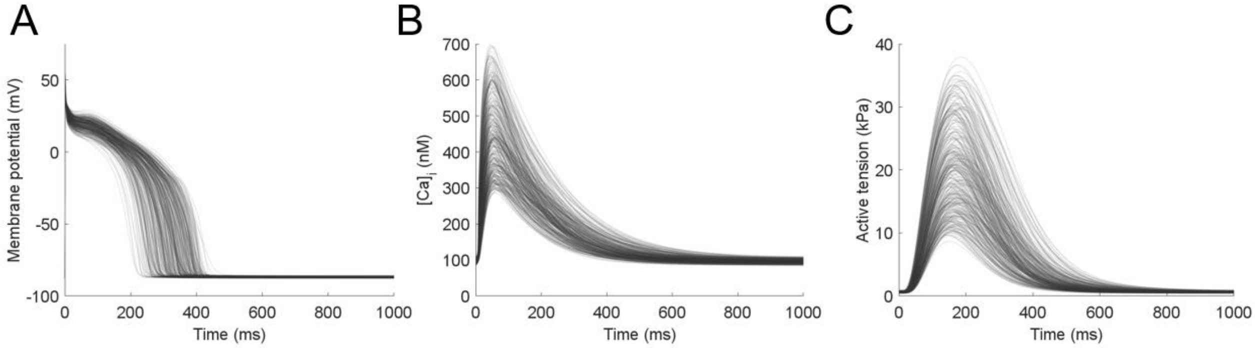

Figure S2. Population of 341 models used in the in silico trial. A) *Action potentials,* ***B)*** *Calcium transients,* ***C)*** *Active tension.*
